# Analysis of factors associated with delayed diagnosis and treatment of testicular torsion in 1005 cases from Chongqing city, China: a cross-sectional study

**DOI:** 10.1038/s41598-023-49820-9

**Published:** 2023-12-20

**Authors:** Hongmei Yi, Delin Wang, Xiaohou Wu, Xiangzhi Gan, Dan Wang, Xin zhao, Honglin Cheng

**Affiliations:** 1 Department of Urology, The First Affiliated Hospital of Chongqing Medical University, Chongqing, 400016 China; 2 Department of Urology, The Affiliated Hospital of Southwest Medical University, Luzhou, 646000 Sichuan China

**Keywords:** Health care, Risk factors, Urology

## Abstract

This study aimed to investigate the clinical and social factors of delayed treatment for testicular torsion (TT) and to explore the risk factors of testicular excision in China. The clinical data of 1005 patients with TT who were admitted to 48 medical institutions in Chongqing city (China) from January 2012 to December 2021 were retrospectively analyzed. It was revealed that the misdiagnosis rates of non-senior (junior and middle) grade doctors and senior doctors were 25.1% and 9.6%, respectively. The proportion of TT patients who received timely treatment (within 6 h after onset of symptoms) was 23.8%. The results of the multivariable logistic regression analysis indicated that absent cremasteric reflex was a protective factor for delayed surgery of more than 6 h from onset of symptoms to surgery. Misdiagnosis, consultation with a non-urologist as the first consultant doctor, absence blood flow in color Doppler ultrasound, negative high-riding testis findings, the presence of fever, and non-manual detorsion were identified as risk factors associated with delayed surgery (more than 6 h from the onset of symptoms) for TT. Furthermore, misdiagnosis, non-urologist first-consultant doctor, absent blood flow in DUS, non-manual detorsion, fever, degree of cord twisting > 180, and the initial diagnosis in tertiary hospitals were risk factors for orchidectomy. Having TT on the right side, and the presence of nausea and vomiting were identified as protective factors for orchidectomy. Technical training in the diagnosis and treatment of TT should be extended to primary hospitals and doctors to significantly improve their accuracy in managing this condition.

## Introduction

Testicular torsion (TT) is a frequent urological surgical emergency characterized by acute scrotal pain. It may be accompanied by additional symptoms, such as fever, testicular swelling, nausea, vomiting, and absent cremasteric reflex^1^. The incidence of TT is approximately 2.02% in South Korea^2^ and 12.6% in Ireland in men aging under 25 years old^3^. Notably, TT is a time-dependent urgent event^4^. Timely diagnosis and surgical intervention of TT are crucial to prevent testicular ischemia and decrease the likelihood of need for orchiectomy secondary to infarction. The two most important determinants of salvage rate of the testis include the degree of cord twisting and the time interval between the onset of symptoms and detorsion^4,5^.

Brazilian scholars demonstrated that 59% of TT patients presented with a delay of more than 6 h^6^. Another pediatric TT study conducted in China found that 73.1% of patients experienced a delay of more than 12 h^5^. Filho et al.^6^ investigated the misdiagnosis rate, inter-hospital transfer time, and testicular salvage in TT patients. Winters et al.^7^ from the UK identified key factors for the acute diagnosis and management of TT. Yu et al.^5^ reported the clinical, socioeconomic, and other factors associated with delayed management of pediatric TT in China. TT has been demonstrated to cause a long-term decrease in sperm motility and reduce overall sperm count^8^. Therefore, timely and accurate diagnosis and treatment are essential for TT patients.

However, there is a lack of multicenter studies concentrating on TT and associated risk factors in China. Therefore, the present cross-sectional study aimed to summarize the clinical manifestations, physical examinations, auxiliary examinations, surgical outcomes, and to identify potential risk factors contributing to testicle removal. This study examined the clinical and social factors associated with delayed surgery and orchidectomy in 48 medical institutions located in Chongqing city (China) between January 2012 and December 2021.

## Participants and methods

### Participants

A retrospective analysis was conducted on 1005 patients with TT from January 2012 to December 2021 in 48 medical institutions in Chongqing city. The inclusion criteria comprised patients diagnosed with TT and who underwent scrotal exploration with detorsion orchiopexy or orchiectomy. Exclusion criteria were patients in whom testicular torsion during scrotal exploration has not been confirmed,with incomplete or missing data. According to each center’s emergency protocol, scrotal Doppler ultrasound (DUS) was requested. The final diagnosis of TT was always made intraoperatively.

### Data collection

All the data were obtained from reliable medical records. Delayed management of TT was defined as a duration exceeding 6 h from the onset of symptoms to surgery, with a specific emphasis on recognizing 24 h as a critical time interval. Additional clinical data included time from the onset of symptoms to surgery, patient’s age, season of onset, first-diagnosed disease, title of the first-consultant doctor, whether the first-consultant doctor is a urology specialist, class of first-consultant hospital, DUS examination, laterality, high riding testis, cremasteric reflex, body temperature, manual detorsion, degree of cord twisting, blood and urine routine examinations, type of torsion, degree of cord twisting, initial symptom and surgical outcomes. Other diagnoses included epididymitis, orchitis, hydrocele, cryptorchidism, acute appendicitis, inguinal hernia, abdominal pain of unknown origin, gastroenteritis, urinary tract stones, and trauma. Clinical symptoms included testicular pain, scrotal pain, abdominal pain, nausea, vomiting, inguinal pain or masses, and fever.

### Statistical analysis

In this study, R 4.1.2 and SPSS 24.0 (IBM, Armonk, NY, USA) software were utilized to carry out statistical analysis. Count data were described as frequency and percentage, and differences between groups were analyzed using the Chi-square test or the Fisher’s exact probability method. Multivariate stepwise logistic regression analysis was employed to identify the influential factors of delayed surgery (more than 6 h from the onset of symptoms to surgery) and orchiectomy. The discriminatory power of the multivariate logistic regression model was evaluated using the area under the receiver operating characteristic (ROC) curve. The significance level was set at α = 0.05, and *P* < 0.05 was considered statistically significant.

### Ethics approval and consent to participate

The present study was approved by the Ethics Committee of the First Affiliated Hospital of Chongqing Medical University (Approval No. K2023-569) and is performed in accordance with the ethical standards laid down in the 1964 Declaration of Helsinki and its later amendments. As the medical records used in this study were obtained from previous diagnoses and treatments, the necessity of the informed consent was waived by the Ethics Committee of the First Affiliated Hospital of Chongqing Medical University. Details that might reveal the study subjects’ identity were excluded.

## Results

**Figure 1:**
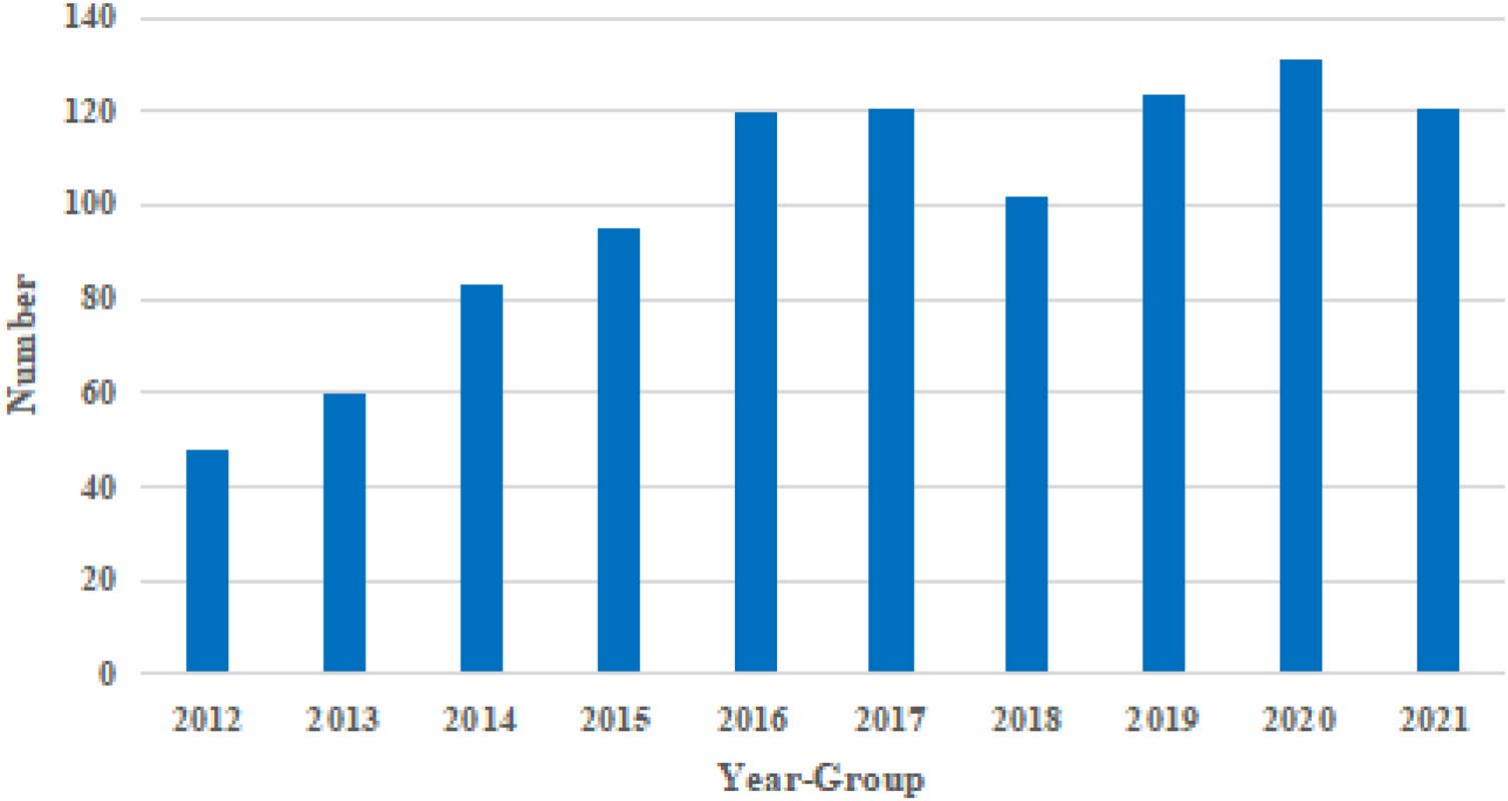
Distribution of annual cases of TT. The number of TT increased year by year from 2012 to 2016, and tended to be stable from 2017 to 2021.

**Figure 2:**
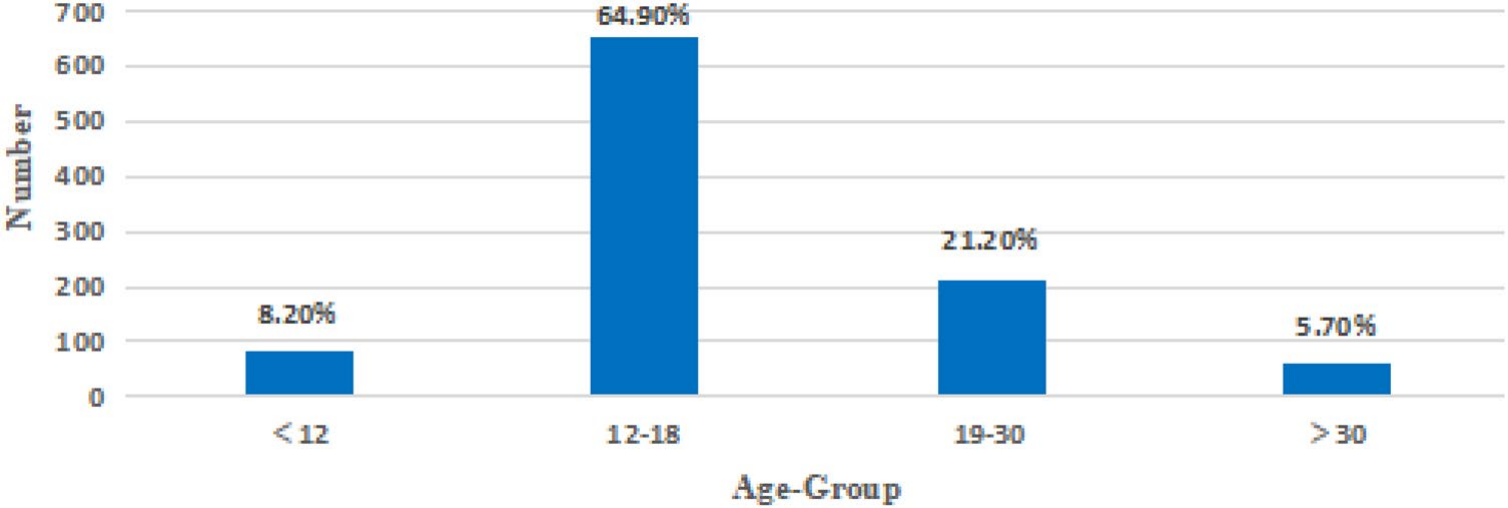
Age distribution of testicular torsion. The peak age for TT is between 12 and 18 years.

**Figure 3:**
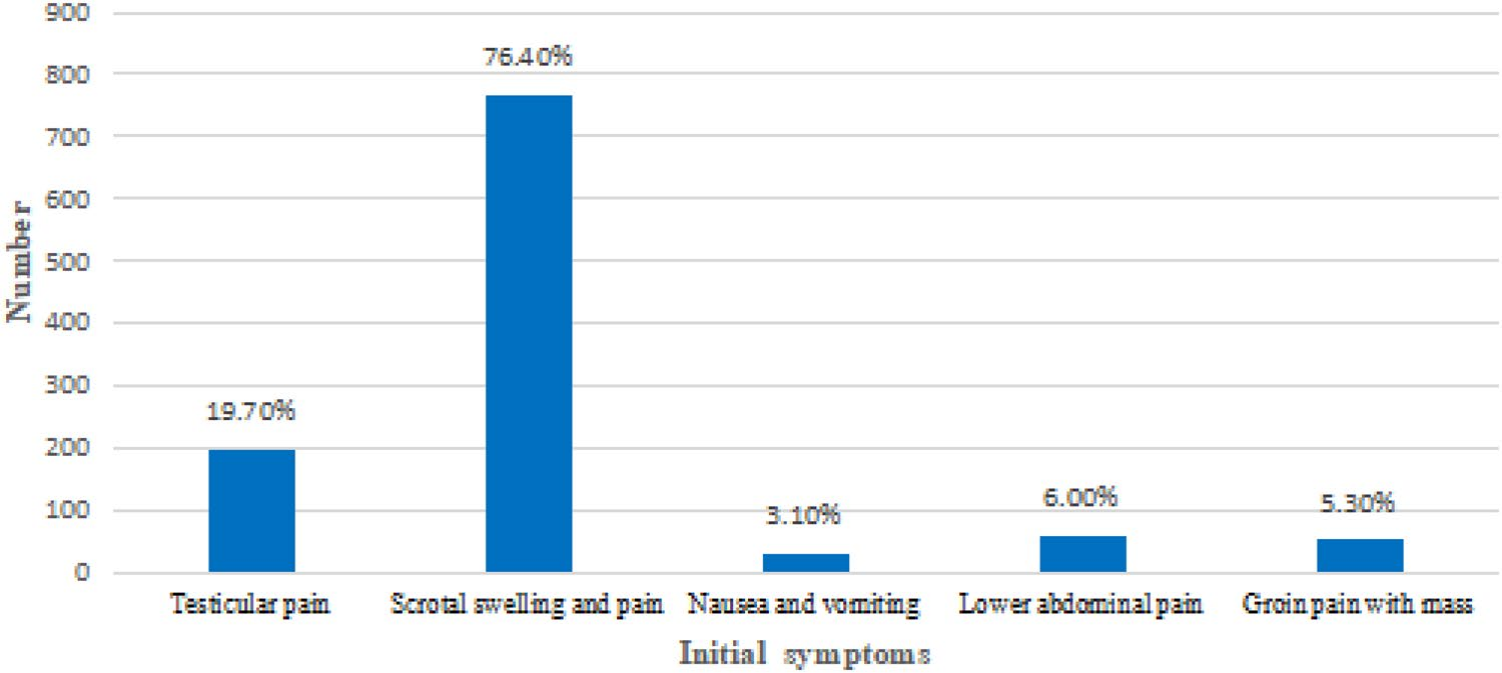
Distribution of initial diagnosis symptoms of TT.

Overall, 1005 men underwent surgical exploration for suspected TT. The number of TT cases increased annually from 2012 to 2016, while it became stable from 2017 to 2021 (Fig. 1). The age group with the highest number of TT surgeries was 12–18 years old (64.9%) (Fig. 2). Scrotal swelling and pain were the primary symptoms (76.4%) (Fig. 3). The results revealed that TT commonly occurred in the spring (29.8%) and winter (33.7%), with an age of onset ranging from 1 to 76 years (17.67 ± 8.51). The overall misdiagnosis rate was 23.0%, with misdiagnosis rates of 37.8% and 11.2% in non-tertiary and tertiary hospitals, respectively. The misdiagnosis rates among non-senior (junior and middle) and senior title were 25.1% and 9.6%, respectively. Approximately 23.8% of TT patients received timely treatment (within 6 h) (Table 1).

**Table 1:** Demographic and clinical characteristics of 1005 TT cases in different duration periods from the onset of symptoms to surgery.

	Total	< 6 h (n = 239)	6–12 h (n = 168)	12–24 h (n = 66)	> 24 h (n = 532)	*P*	*P*^a^	*P*^b^	*P*^c^
Age (years)						0.027	0.058	0.003	0.001
< 12	82(8.1)	11(4.6)	10(6.0)	4(6.1)	57(10.7)				
12–18	652(64.9)	158(66.1)	100(59.5)	45(68.2)	349(65.6)				
19–30	213(21.2)	59(24.7)	45(26.8)	15(22.7)	94(17.7)				
> 30	58(5.8)	11(4.6)	13(7.7)	2(3.0)	32(6.0)				
Season						0.110	0.108	0.852	0.671
Spring	299(29.8)	69(28.9)	54(32.1)	13(19.7)	163(30.7)				
Summer	183(18.2)	35(14.6)	34(20.3)	16(24.2)	98(18.4)				
Autumn	184(18.3)	55(23.0)	22(13.1)	17(25.8)	90(16.9)				
Winter	339(33.7)	80(33.5)	58(34.5)	20(30.3)	181(34.0)				
First-diagnosed disease						< 0.001	< 0.001	< 0.001	< 0.001
Testicular torsion	774(77.0)	233(97.5)	147(87.5)	57(86.4)	337(63.4)				
Misdiagnosis	231(23.0)	6(2.5)	21(12.5)	9(13.6)	195(36.6)				
Whether the first-consultan doctor is a urology specialist						< 0.001	< 0.001	< 0.001	< 0.001
Yes	699(69.6)	210(87.9)	126(75.0)	47(71.2)	316(59.4)				
No	306(30.4)	29(12.1)	42(25.0)	19(28.8)	216(40.6)				
Testicular blood flow on color Doppler ultrasound						< 0.001	< 0.001	< 0.001	< 0.001
Decreased	424(42.2)	134(56.1)	95(56.5)	39(59.1)	156(29.3)				
Absent	552(54.9)	101(42.2)	68(40.5)	25(37.9)	358(67.3)				
Preserved blood flow	29(2.9)	4(1.7)	5(3.0)	2(3.0)	18(3.4)				
Laterality						0.285	0.103	0.082	0.095
Left	619(61.8)	136(56.9)	101(60.5)	41(63.1)	341(64.2)				
Right	383(38.2)	103(43.1)	66(39.5)	24(36.9)	190(35.8)				
High riding testis						0.026	0.012	0.006	0.007
Positive	841(83.7)	213(89.1)	144(85.7)	55(83.3)	429(80.6)				
Negative	164(16.3)	26(10.9)	24(14.3)	11(16.7)	103(19.4)				
Cremasteric reflex						0.243	0.701	1.000	0.380
Positive	526(52.3)	122(51.0)	91(54.2)	42(63.6)	271(50.9)				
Negative	479(47.7)	117(49.0)	77(45.8)	24(36.4)	261(49.1)				
Urine routine examination						0.144	0.094	0.046	0.154
Positive	73(7.3)	11(4.6)	10(6.0)	7(10.6)	45(8.5)				
Negative	932(92.7)	228(95.4)	158(94.0)	59(89.4)	487(91.5)				
Blood routine examination						0.238	0.182	0.091	0.331
Normal	557(55.4)	123(51.5)	89(53.0)	42(63.6)	303(57.0)				
Elevated	448(44.6)	116(48.5)	79(47.0)	24(36.4)	229(43.0)				
Body temperature						0.003	0.001	0.001	0.005
Normal	920(91.5)	232(97.1)	155(92.3)	59(89.4)	474(89.1)				
Fever	85(8.5)	7(2.9)	13(7.7)	7(10.6)	58(10.9)				
Manual detorsion						< 0.001	0.001	0.001	< 0.001
Yes	96(9.6)	37(15.5)	18(10.7)	9(13.6)	32(6.0)				
No	909(90.4)	202(84.5)	150(89.3)	57(86.4)	500(94.0)				
Orchiectomy or not						< 0.001	< 0.001	< 0.001	< 0.001
Yes	532(52.9)	14(5.9)	46(27.4)	35(53.0)	437(82.1)				
No	473(47.1)	225(94.1)	122(72.6)	31(47.0)	95(17.9)				
Type of torsion						0.836	0.410	0.365	0.299
Intra-vaginal	611(60.8)	154(64.4)	104(61.9)	41(62.1)	312(58.6)				
Extra-vaginal	341(33.9)	73(30.5)	55(32.7)	21(31.8)	192(36.1)				
Between testis and epididymis	53(5.27)	12(5.02)	9(5.36)	4(6.06)	28(5.26)				
Degree of cord twisting						< 0.001	0.055	< 0.001	< 0.001
0–180°	212(21.1)	64(26.8)	54(32.1)	9(13.7)	85(16.0)				
181°–360°	427(42.5)	104(43.5)	66(39.3)	28(42.4)	229(43.0)				
361°–540°	165(16.4)	30(12.6)	17(10.1)	14(21.2)	104(19.5)				
541°–720°	172(17.1)	34(14.2)	28(16.7)	14(21.2)	96(18.0)				
> 720°	29(2.9)	7(2.9)	3(1.8)	1(1.5)	18(3.38)				
Title of the first-consultan doctor									
Junior and middle	869	206	139	52	472	< 0.001	< 0.001	< 0.001	< 0.001
Testicular torsion	651(74.9)	200(97.1)	119(85.6)	43(82.7)	289(61.2)				
Misdiagnosis	218(25.1)	6(2.9)	20(14.4)	9(17.3)	183(38.8)				
Senior	136	33	29	14	60	0.003	0.038	0.010	0.001
Testicular torsion	123(90.4)	33(100)	28(96.6)	14(100)	48(80.0)				
Misdiagnosis	13(9.6)	0(0.00)	1(3.45)	0(0.00)	12(20.0)				
Class of first-consultant hospital									
Primary and secondary health-care unit	444	92	72	26	254	< 0.001	< 0.001	< 0.001	< 0.001
Testicular torsion	276(62.2)	91(98.9)	58(80.6)	18(69.2)	109(42.9)				
Misdiagnosis	168(37.8)	1(1.1)	14(19.4)	8(30.8)	145(57.1)				
Tertiary health-care unit	561	147	96	40	278	< 0.001	0.001	< 0.001	< 0.001
Testicular torsion	498(88.8)	142(96.6)	89(92.7)	39(97.5)	228(82.0)				
Misdiagnosis	63(11.2)	5(3.4)	7(7.3)	1(2.5)	50(18.0)				
Testicular pain						0.856	0.525	0.959	0.960
No	807(80.3)	188(78.7)	138(82.1)	53 (80.3)	428(80.5)				
Yes	198(19.7)	51 (21.3)	30 (17.9)	13 (19.7)	104(19.5)				
Scrotal swelling and pain						0.872	0.881	0.498	0.547
No	237(23.6)	55 (23.0)	36 (21.4)	16 (24.2)	130(24.4)				
Yes	768(76.4)	184(77.0)	132 (78.6)	50 (75.8)	402(75.6)				
Nausea and vomiting						0.023	> 0.999	0.143	0.031
No	974(96.9)	232(97.1)	158 (94.0)	62 (93.9)	522(98.1)				
Yes	31 (3.08)	7 (2.93)	10 (5.95)	4 (6.06)	10 (1.88)				
Lower abdominal pain						0.375	0.239	0.193	0.465
No	945(94.0)	229(95.8)	159 (94.6)	60 (90.9)	497(93.4)				
Yes	60 (6.0)	10 (4.2)	9 (5.4)	6 (9.1)	35 (6.6)				
Groinpain with mass						0.103	0.486	0.254	1.000
No	952(94.7)	229(95.8)	161 (95.8)	58 (87.9)	504 (94.7)				
Yes	53 (5.3)	10 (4.2)	7 (4.2)	8 (12.1)	28 (5.3)				

^a^*P* < 6 h vs ≥ 6 h; ^b^*P* < 12 h vs ≥ 12 h; ^c^*P* < 24 h vs ≥ 24 h.

Significant differences (*P* < 0.05) were found in various baseline factors among the four groups based on the surgical time interval from the onset of symptoms to surgery (< 6 h, 6–12 h, 12–24 h, and > 24 h). These factors included age, first-diagnosed disease, whether the first-consultant doctor was a urology specialist, DUS examination, high riding testis, urine routine, body temperature, manual detorsion, whether the testicle was removed, degree of cord twisting, first-consultant doctor’s title, class of first-consultant hospital, nausea, and vomiting. No significant differences (*P* > 0.05) were identified in season, laterality, cremasteric reflex, blood routine, type of torsion, testicular pain, scrotal swelling and pain, lower abdominal pain, and groin pain with mass (Table 1).

The variables summarized in Table 1 were involved in stepwise logistic regression analysis as independent variables with the dependent variable that was delayed surgery for more than 6 h after the onset of symptoms. The results indicated that the absent cremasteric reflex was a protective factor for delayed surgery (> 6 h from the onset of symptoms). Misdiagnosis, consultation with a non-urologist as the first consultant doctor, absence blood flow in DUS, negative high-riding testis findings, the presence of fever, and non-manual detorsion were identified as risk factors associated with delayed surgery (more than 6 h from the onset of symptoms) for TT) (Table 2).

**Table 2:** Multivariable logistic regression analysis of risk factors associated with delayed surgical management for TT (more than 6 h from onset of symptoms to surgery).

Variable	β	SE	Z	OR(95%CI)	*P*
First-diagnosed disease					
Testicular torsion				Ref	
Misdiagnosis	2.296	0.431	5.325	9.93(4.632,25.848)	< 0.001
Whether the first-consultant doctor is a urology specialist					
Yes				Ref	
No	0.990	0.231	4.284	2.692(1.733,4.301)	< 0.001
Testicular blood flow on color doppler ultrasound					
Decreased				Ref	
Absent	0.676	0.164	4.121	1.967(1.427,2.717)	< 0.001
Preserved blood flow	0.823	0.583	1.413	2.278(0.793,8.25)	0.158
Cremasteric reflex					
Positive				Ref	
Negative	− 0.503	0.172	− 2.924	0.605(0.431,0.847)	0.003
High riding testis					
Positive				Ref	
Negative	0.733	0.258	2.839	2.081(1.270,3.505)	0.005
Body temperature					
Normal				Ref	
Fever	1.174	0.416	2.821	3.236(1.525,7.987)	0.005
Manual detorsion					
Yes				Ref	
No	0.587	0.246	2.381	1.798(1.104,2.909)	0.017

*SE* Standard error of the parameter estimate; *OR* Odds ratio, *CI* Confidence interval.

Statistically significant differences (*P* < 0.05) were found between the orchiectomy and orchiopexy groups in several factors. These factors included the first-diagnosed disease, whether the first-consultant doctor was a urology specialist, color DUS examination, laterality, high riding testis, body temperature, manual detorsion, scrotal swelling and pain, and degree of cord twisting. There were no statistically significant differences in age, season, cremasteric reflex, urine routine, blood routine, the title of the first-consultant, class of the first-consultant hospital, type of torsion, testicular pain, nausea and vomiting, lower abdominal pain, and groin pain with mass (*P* > 0.05) (Table 3).

**Table 3:** Patient characteristics and risk factors of orchidectomy.

Variable	Total (n = 1005)	Orchidectomy (n = 532)	Orchidopexy (n = 473)	*P*	χ^2^
Age (years)				0.256	4.051
< 12	82(8.1)	42(7.9)	40(8.4)		
12–18	652(64.9)	355(66.7)	297(62.8)		
19–30	213(21.2)	101(19.0)	112(23.7)		
> 30	58(5.8)	34(6.4)	24(5.1)		
Season				0.773	1.117
Spring	299(29.8)	155(29.1)	144(30.4)		
Summer	183(18.2)	103(19.4)	80(16.9)		
Autumn	184(18.3)	98(18.4)	86(18.2)		
Winter	339(33.7)	176(33.1)	163(34.5)		
First-diagnosed disease				< 0.001	44.116
Testicular torsion	774(77.0)	365(68.6)	409(86.5)		
Misdiagnosis	231(23.0)	167(31.4)	64(13.5)		
Whether the first-consultant doctor is a urology specialist				< 0.001	23.791
Yes	699(69.6)	334(62.8)	365(77.2)		
No	306(30.4)	198(37.2)	108(22.8)		
Testicular blood flow on color Doppler ultrasound				< 0.001	104.277
Decreased	424(42.2)	153(28.8)	271(57.3)		
Absent	552(54.9)	372(69.9)	180(38.1)		
Preserved blood flow	29(2.9)	7(1.3)	22(4.6)		
Laterality				0.005	8.046
Left	619(61.6)	350(65.8)	269(56.9)		
Right	386(38.4)	182(34.2)	204(43.1)		
High riding testis				0.012	6.308
Positive	841(83.7)	430(80.8)	411(86.9)		
Negative	164(16.3)	102(19.2)	62(13.1)		
Cremasteric reflex				0.315	1.009
Positive	526(52.3)	270(50.8)	256(54.1)		
Negative	479(47.7)	262(49.2)	217(45.9)		
Urine routine examination				0.348	0.882
Positive	73(7.3)	43(8.1)	30(6.3)		
Negative	932(92.7)	489(91.9)	443(93.7)		
Blood routine examination				0.331	0.946
Normal	557(55.4)	303(57.0)	254(53.7)		
Elevated	448(44.6)	229(43.0)	219(46.3)		
Body temperature				< 0.001	14.052
Normal	920(91.5)	470(88.3)	450(95.1)		
Fever	85(8.5)	62(11.7)	23(4.9)		
Manual detorsion				< 0.001	27.336
Yes	96(9.6)	26(4.9)	70(14.8)		
No	909(90.4)	506(95.1)	403(85.2)		
Type of torsion				0.050	5.996
Intra-vaginal	611(60.8)	312(58.6)	299(63.2)		
Extra-vaginal	341(33.9)	197(37.0)	144(30.4)		
Between testis and epididymis	53(5.27)	23(4.32)	30(6.34)		
Degree of cord twisting				< 0.001	148.105
0–180°	212(21.1)	40(7.5)	172(36.4)		
181°–360°	427(42.5)	232(43.6)	195(41.2)		
361°–540°	165(16.4)	120(22.6)	45(9.5)		
541°–720°	172(17.1)	118(22.2)	54(11.4)		
> 720°	29(2.9)	22(4.1)	7(1.5)		
Class of first-consultant hospital				0.953	0.004
Primary and secondary health-care unit	444(44.2)	236(44.4)	208(44.0)		
Tertiary health-care unit	561(55.8)	296(55.6)	265(56.0)		
Title of the first-consultant doctor				0.407	0.689
Junior and middle	869(86.5)	465(87.4)	404(85.4)		
Senior	136(13.5)	67(12.6)	69(14.6)		
Testicular pain				0.063	3.449
No	807(80.3)	415 (78.0)	392 (82.9)		
Yes	198(19.7)	117 (22.0)	81 (17.1)		
Scrotal swelling and pain				0.037	4.371
No	237(23.6)	140 (26.3)	97 (20.5)		
Yes	768(76.4)	392 (73.7)	376 (79.5)		
Nausea and vomiting				0.287	1.131
No	974(96.9)	519 (97.6)	455 (96.2)		
Yes	31 (3.1)	13 (2.4)	18 (3.8)		
Lower abdominal pain				0.072	3.231
No	945(94.0)	493 (92.7)	452 (95.6)		
Yes	60 (6.0)	39 (7.3)	21 (4.4)		
Groinpain with mass				0.660	0.193
No	952(94.7)	506 (95.1)	446 (94.3)		
Yes	53 (5.3)	26 (4.9)	27 (5.7)		

The variables summarized in Table 3 were involved in stepwise logistic regression analysis as independent variables with the dependent variable that was orchidectomy. The results revealed that misdiagnosis, non-urologist first-consultant doctor, absent blood flow in DUS, non-manual detorsion, fever, degree of cord twisting > 180, and the initial diagnosis in tertiary hospitals were risk factors for orchidectomy. Having TT on the right side, and the presence of nausea and vomiting were identified as protective factors for orchidectomy (Table 4).

**Table 4:** Multivariate logistic regression analysis of risk factors for orchiectomy.

Variable	β	SE	Z	OR(95%CI)	*P*
First-diagnosed disease					
Testicular torsion				Ref	
Misdiagnosis	1.028	0.215	4.787	2.795(1.844,4.283)	< 0.001
Whether the first-consultant doctor is a urology specialist					
Yes				Ref	
No	0.575	0.194	2.972	1.778(1.218,2.604)	0.003
Testicular blood flow on color Doppler ultrasound					
Decreased				Ref	
Absent	1.157	0.155	7.461	3.179(2.350,4.318)	< 0.001
Preserved blood flow	− 0.403	0.502	− 0.802	0.668(0.236,1.728)	0.423
Laterality					
Left				Ref	
Right	− 0.337	0.156	− 2.157	0.714(0.525,0.969)	0.031
Body temperature					
Normal				Ref	
Fever	0.938	0.305	3.075	2.555(1.427,4.732)	0.002
Manual detorsion					
Yes				Ref	
No	1.389	0.272	5.100	4.009(2.38,6.941)	< 0.001
Degree of cord twisting					
0–180°				Ref	
181°–360°	1.652	0.218	7.580	5.219(3.434,8.083)	< 0.001
361°–540°	2.434	0.275	8.847	11.409(6.722,19.796)	< 0.001
541°–720°	2.299	0.267	8.616	9.963(5.961,16.988)	< 0.001
> 720°	2.257	0.510	4.429	9.553(3.662,27.551)	< 0.001
Class of first-consultant hospital					
Junior and middle				Ref	
Senior	0.576	0.170	3.381	1.778(1.277,2.489)	0.001
Nausea and vomiting					
No				Ref	
Yes	− 0.987	0.433	− 2.282	0.373(0.157,0.868)	0.022

*SE* Standard error of the parameter estimate; *OR* Odds ratio; *CI *Confidence interval.

## Discussion

To date, few studies have concentrated on TT worldwide, and no large-scale study has explored the risk factors for orchidectomy in China. The present study investigated the clinical, social, and diagnostic statuses of delayed surgical management that may lead to TT. The definition of delayed surgical intervention for TT remains elusive. The latest 2022 EAU Pediatric Urology Guidelines demonstrated that early surgical intervention (mean torsion time shorter than 13 h) with detorsion was found to preserve fertility, and TT symptoms occurring after 6 h may cause irreversible changes^9^. The present study revealed that delayed treatment of TT was directly associated with testicular salvage ability. As the time from symptom onset to treatment extended, the rate of orchidectomy exhibited a noticeably upward trend. The orchidectomy rate was 5.9% in patients who underwent surgery within 6 h after onset of symptoms, while it rose to 82.1% in patients who underwent surgery at 24 h after onset of symptoms. Only 23.8% of TT patients received timely treatment within 6 h after onset of symptoms. Therefore, as a time-sensitive diagnosis, early recognition and definitive management of TT are critical^10^.

In the present study, the peak age of TT patients in Chongqing city was 12–18 years old, accounting for 64.9%, followed by 19–30 years old, accounting for 21.2%, which is similar to previous research results^11^. Testicular loss in these two age groups may seriously affect patients’ fertility^12^. In terms of seasonality, the present study indicated that the incidence rate was higher in cold seasons, with the highest incidence rate in winter, followed by in spring, which is consistent with Scotland et al.’s^13^ and Takeshita et al.’s^14^ findings. The mechanism may be related to the strong contraction of the scrotum in cold seasons, and a hyperactive cremasteric reflex stimulated by cold weather has appeared as a precipitating factor.

The results of the present study showed that the misdiagnosis rates in non-tertiary (primary and secondary) and tertiary hospitals were 37.8% and 11.2%, respectively, and the misdiagnosis rates among non-senior (primary and intermediate) grade and senior title doctors were 25.1% and 9.6%, respectively. This could be attributed to the lack of specialized training received by doctors with lower hospital grades and professional titles. Therefore, it is essential to strengthen the specialized training of doctors in primary hospitals and improve their accuracy in diagnosis and treatment. A study conducted in Brazil concluded that the highest testicular salvage rate was in tertiary hospitals, which is consistent with the results of the present study^6^. Kumar et al.^15^ and Ogbetere et al.^16^ demonstrated that a greater effort to educate primary health care professionals may reduce this delay. Small/medium community hospitals exhibited the lowest orchiectomy rates in Canada, and longer ER throughput was consistently associated with loss of the testicle^17^, which could be related to the higher level of medical care in developed countries. However, in the present study, the multivariate logistic regression analysis indicated that tertiary hospitals were a risk factor for orchidectomy. The potential reasons for this discrepancy could be attributed to the extended travel distance that patients must cover to reach a tertiary hospital as their initial consulting facility, resulting in delayed treatment. Alternatively, it might be due to insufficient attention from the patient or their parents. The study did not collect data on the patient’s residence and reasons for delayed treatment, necessitating further research.

In the present study, the results of the multivariate logistic regression analysis indicated that absent cremasteric reflex was a protective factor for delayed surgery for more than 6 h from onset of symptoms to surgery. Absent cremasteric reflex was found as a protective factor for delayed surgery for more than 6 h from onset of symptoms to surgery, because it assisted doctors to accurately diagnose TT, and shortened the time to diagnosis^18^. A negative high riding testis was identified as a risk factor for delayed surgery for more than 6 h from the onset of symptoms to surgery, which is consistent with previously reported findings^19,20^.

The results of the multivariate logistic regression analysis in the present study demonstrated that the misdiagnosis, non-urologist first-consultant doctor, absent blood flow in DUS, non-manual detorsion, fever, and degree of cord twisting  > 180 were risk factors for orchidectomy. Nausea and vomiting, and right-sided TT were protective factors for orchidectomy. Misdiagnosing a non-TT condition as the primary diagnosis can result in the prolonged transfer time and delayed treatment^6^. The clinical symptoms of TT encompass not only testicular pain and scrotal swelling, but also nausea and vomiting, lower abdominal pain, and a palpable lump in the inguinal region. Seeking consultation from a non-urology specialist as the initial healthcare provider increases the likelihood of misdiagnosis and treatment delay.

Pinar et al.^21^ assessed 2922 TT cases, and a strong sensitivity of 85.2% and a specificity of 52.7% were achieved using DUS. If DUS reveals a loss of blood flow, it signifies prolonged ischemia in the patient’s testicle, reducing the likelihood of testicular salvage and increasing the risk of orchidectomy. Scrotal exploration should be promptly conducted, even though testicular blood flow is observed, when there is suspicion of TT based on medical history and physical examination findings^22^. Manual detorsion can alleviate ischemia, providing immediate symptom relief. Li et al.^23^ demonstrated that manual detorsion may contribute to a timely increase in the rate of testicular salvage before emergency surgery.

Manivel et al.^23^ concluded that training emergency physicians in scrotal ultrasound and manual detorsion of a twisted testicle can reduce the time to diagnosis and reperfusion. Their findings suggested that fever is a risk factor for TT, with elevated patient’s body temperature indicating advanced disease and more severe ischemic necrosis of the testicles, leading to a higher removal rate. The current study presented fewer cases of torsional fever. Numerous studies have established a correlation between the degree of testicular twist and the risk of a non-salvageable testis^25–27^. A more remarkable degree of TT is associated with more severe ischemia, making it a risk factor for orchidectomy^25–27^.

Nausea and vomiting were found as protective factors for orchidectomy, which is contrary to the findings of a previous study on adult TT patients^28^. This difference could be attributed to the higher percentage (73.03%) of minors in the current study population, promoting the detection of the condition when patients experienced nausea and vomiting, leading to seeking timely medical attention.

The present study adds to the limited number of large-scale studies on TT in both adults and children in China. However, several questions remain unanswered, and future research should aim to address these concerns to enhance the robustness and comprehensiveness of findings in the field of TT. Firstly, retrospective studies may have recall bias and selection bias. Secondly, due to the lack of database, manually collecting data from multiple centers may result in incomplete data, and the predicted incidence rate may be lower than the actual incidence rate. Thirdly, certain factors contributing to delayed surgery, such as medical insurance, family economic status, medical level of residence and distance from the hospital, guardian’s or patient’s educational level, were not sufficiently captured and require further assessment. Finally, no follow-up study was conducted on the fertility and hormone levels of patients who underwent orchidectomy and orchidopexy, which will be performed in the future research.

## Conclusions

In this multicenter retrospective study, the misdiagnosis rate of TT in Chongqing city was found to be 23.0%, and higher rates were noted among non-senior title doctors and non-tertiary hospitals. Only about 23.8% of TT patients received timely treatment. Misdiagnosis, non-urologist first-consultant doctor, absent blood flow in DUS, non-manual detorsion, fever, degree of cord twisting  > 180, and the initial diagnosis in tertiary hospitals were identified as risk factors for orchidectomy. It is essential to strengthen the training programs for the diagnosis and treatment of TT for doctors in primary hospitals, lower professional titles, and emergency departments to improve their abilities of accurate diagnosis and treatment, so as to increase the rate of testicular salvage.

## Data Availability

The datasets used and/or analyzed during the current study are available from the corresponding author upon reasonable request.

## Abbreviation

TT: Testicular torsion
